# Using machine learning-based analysis for behavioral differentiation between anxiety and depression

**DOI:** 10.1038/s41598-020-72289-9

**Published:** 2020-10-02

**Authors:** Thalia Richter, Barak Fishbain, Andrey Markus, Gal Richter-Levin, Hadas Okon-Singer

**Affiliations:** 1 Department of Psychology, School of Psychological Sciences, University of Haifa, Mount Carmel, 3498838 Haifa, Israel; 2 Faculty of Civil and Environmental Engineering, Technion-Israel Institute of Technology, Haifa, Israel

**Keywords:** Human behaviour, Computer science

## Abstract

Anxiety and depression are distinct—albeit overlapping—psychiatric diseases, currently diagnosed by self-reported-symptoms. This research presents a new diagnostic methodology, which tests rigorously for differences in cognitive biases among subclinical anxious and depressed individuals. 125 participants were divided into four groups based on the levels of their anxiety and depression symptoms. A comprehensive behavioral test battery detected and quantified various cognitive–emotional biases. Advanced machine-learning tools, developed for this study, analyzed these results. These tools detect unique patterns that characterize anxiety versus depression to predict group membership. The prediction model for differentiating between symptomatic participants (i.e., high symptoms of depression, anxiety, or both) compared to the non-symptomatic control group revealed a 71.44% prediction accuracy for the former (sensitivity) and 70.78% for the latter (specificity). 68.07% and 74.18% prediction accuracy was obtained for a two-group model with high depression/anxiety, respectively. The analysis also disclosed which specific behavioral measures contributed to the prediction, pointing to key cognitive mechanisms in anxiety versus depression. These results lay the ground for improved diagnostic instruments and more effective and focused individually-based treatment.

## Introduction

The vast overlap between anxiety and depression is manifested in many areas, including comorbidity rates^1^, physical and emotional symptoms^2^, diagnostic questionnaires^3^, risk factors such as related genes and negative life events^4^, the prescription of similar medications and common psychological therapies^2,5^. The existing self-reported symptoms-based diagnosis method is limited in its ability to detect specific differences between the disorders^6^. Innovative diagnostic paradigms are needed to advance our understanding and lead to more accurate treatment methods. In the current study, we present a method that may be used alongside a clinical interview implemented by a psychiatrist, designed to increase the clinician’s objectivity regarding and confidence in the diagnosis.

The current study sought to examine the possibility of differentiating between anxiety and depression by detecting a unique pattern of biased reactions to emotional stimuli that characterize each disorder. This unique characterization is based on participants’ aggregated performance in several behavioral tasks, which target different cognitive functions known to be abnormal in anxiety and/or depression, and does not rely on self-report measures. Biased reactions to emotional stimuli appear in many previous studies among depressed and anxious individuals (e.g.,^7–10^), and are indicators of biased processing of the environment in such populations. These biases maintain disordered affect and play a key role (either causal or contributory) in the onset, maintenance and possibility of recovery from these disorders. Cognitive biases, however, are only one factor out of many explaining the differences among these disorders and, therefore, alone cannot be used to differentiate among them. Rather, adding a diagnostic method based on cognitive biases to the diagnostic process may be of great assistance, as, for instance, is currently done with the Test of Variables of Attention (TOVA) for the diagnosis of ADHD^11^. Currently, the self-reported symptom-based interview cannot be replaced, but it may benefit notably from the addition of objective scores. The results of using this tool together with the self-reported diagnosis and the clinical interview may be increased diagnostic specificity and precision, leading to a more fine-tuned individually-tailored therapy in the future.

To accomplish our objective, a comprehensive test battery was developed. The battery focuses on four of the most investigated bias categories: (a) attention biases: distinct sensitivity to threatening stimuli in the environment and difficulty in disregarding emotional distractors^12–14^; (b) memory biases: enhanced remembering of content that is related to the disorder, relative to other information that was previously coded^15^; (c) interpretation biases: a tendency to interpret ambiguous stimuli as threatening or negative to the self^16^; and (d) expectancy biases: the tendency to expect an increased probability of negative events^17^.

Each bias is examined by a prevalent paradigm targeting this specific function. In addition, given that the literature suggests that differences between anxiety and depression are related to the level of automaticity of cognitive biases^18,19^, each task was modified to test both automatic and non-automatic reactions. Cognitive control ability was also measured, since it was found deficient among depressed and anxious individuals, and linked to their cognitive biases^20,21^.

To detect a behavioral pattern for each group, machine-learning analysis tools were employed. These tools allow detection of complex non-linear high-dimensional interactions that may inform predictions, even in the presence of major instrumental and scoring noise^22^. Specifically, machine-learning methods based on decision tree algorithms were used. They were designed to be sufficiently sensitive for classification of participants into four groups—high anxiety and low depression levels [HA]; high depression and low anxiety levels [HD]; high anxiety and depression levels [HAD]; low anxiety and depression levels [LAD]). A symptomatic profile was formulated for each participant by labeling his or her anxiety and depression levels using the responses on the self-report questionnaires. The machine-learning method was trained to infer each participant’s symptomatic profile based on the behavioral tasks.

We expected that knowledge derived from this systematic investigation would enable us to map and differentiate the unique pattern of biases characterizing each disorder, as well as provide knowledge regarding the characteristics of normative affective processing. Additionally, for each disorder, this knowledge was expected to facilitate a systematic and comprehensive examination of the relationship between symptom level and specific cognitive deficiencies.

## Methods

### Participants

126 native Hebrew speakers with normal or corrected-to-normal eyesight took part in the study. One participant did not finish the battery due to noncompliance with instructions, resulting in a total sample size of 125 participants. The study was performed in accordance with the Declaration of Helsinki. All study methods were performed in accordance with the relevant guidelines and regulations. The experimental protocol was approved by the ethics committee of the Department of Psychology of the University of Haifa. Participants signed an informed consent form prior to participation and were debriefed at the end of the experiment.

Standard power analyses, as done using ANOVA, cannot be done in machine-learning based analyses, which are not based on a distribution of a single factor in different populations. Classification is typically measured either by confusion matrices^23,24^, or through a comparison of the classification accuracy with respect to random clustering^25,26^. In this work we present both criteria.

Table 1 provides demographic and clinical information by group, and the “Supplementary Material” adds further data.

**Table 1:** Mean and standard deviations (in parenthesis) of demographic and clinical information by group.

Group	HA (n = 20)	HD (n = 21)	HAD (n = 18)	LAD (n = 66)
**Age**	26 (8.68)	26 (4.14)	29 (8.18)	25 (4.84)
HA		n.s	n.s	n.s
HD	n.s		n.s	n.s
HAD	n.s	n.s		p < 0.5
LAD	n.s	n.s	p < 0.05	
Sex	17 women (85%)	18 women (85%)	13 women (68%)	48 women (72%)
**Education (years)**	13 (1.92)	14 (2.80)	13 (1.72)	13 (2.25)
HA				
HD	n.s	n.s	n.s	n.s
HAD	n.s	n.s	n.s	n.s
LAD	n.s	n.s	n.s	n.s
**BDI score**	12 (9.10)	17 (9.70)	15 (8.77)	7 (7.24)
HA		n.s	n.s	p < 0.05
HD	n.s		n.s	p < 0.05
HAD	n.s	n.s		p < 0.05
LAD	p < 0.05	p < 0.05	p < 0.05	
**STAI score**	47 (11.26)	52 (9.91)	51 (7.86)	37 (9.82)
HA		n.s	n.s	p < 0.05
HD	n.s		n.s	p < 0.05
HAD	n.s	n.s		p < 0.05
LAD	p < 0.05	p < 0.05	p < 0.05	
**PSWQ score**	58 (11.31)	61 (10.35)	57 (10.30)	47 (12.19)
HA		n.s	n.s	p < 0.05
HD	n.s		n.s	p < 0.05
HAD	n.s	n.s		p < 0.05
LAD	p < 0.05	p < 0.05	p < 0.05	
**RRS score**	49 (14.75)	51 (14.41)	51 (12.91)	38 (13.8)
HA		n.s	n.s	p <0 .05
HD	n.s		n.s	p < 0.05
HAD	n.s	n.s		p <0 .05
LAD	p < 0.05	p < 0.05	p < 0.05	
**DASS depression score**	14 (4.84)	28 (6.53)	27 (4.05)	6 (6.27)
HA		p < 0.05	p < 0.05	
HD	p < 0.05		n.s	p < 0.05
HAD	p < 0.05	n.s		p < 0.05
LAD	p < 0.05	p < 0.05	p < 0.05	p < 0.05
**DASS anxiety score**	20 (4.08)	8 (3.21)	22 (4.71)	4 (4.24)
HA		p < 0.05	n.s	
HD	p < 0.05		p < 0.05	p < 0.05
HAD	n.s	p < 0.05		p < 0.05
LAD	p < 0.05	p < 0.05	p < 005	p < 0.05
**DASS stress score**	24 (8.40)	22 (9.73)	30 (8.09)	11 (9.95)
HA		n.s	n.s	
HD	n.s		p < 0.05	p < 0.05
HAD	n.s	p < 0.05		p < 0.05
LAD	p < 0.05	p < 0.05	p < 0.05	p < 0.05

Participants were recruited from the University of Haifa through advertisements posted around campus, the SONA subject pool system and posting to relevant social media groups. Responders were initially screened by the Depression Anxiety Stress Scales-21 (DASS-21; see elaboration in the questionnaire section below). Participants who scored between 0 and 20 on the depression scale and between 0 and 14 on the anxiety scale of the DASS-21 questionnaire were considered to be showing normal to moderate severity of depression and anxiety according to the recommended cut-off scores^3^. These participants were assigned to the control group (LAD). Participants who scored 21 or more on the depression scale and/or 15 or more on the anxiety scale were considered as having severe to extremely severe anxiety and/or depression traits and were assigned to one of the three experiment groups (i.e., HA, HD, HAD). To control for factors that might interfere with the results, participants were asked about their neurological and psychiatric history (epilepsy or hemophilia conditions, past and current depression/anxiety diagnoses and related prescribed medications, or any other psychiatric or neurological history, syndromes or diseases), as well as the presence of any learning disabilities or attention deficit disorders. Participants received monetary compensation for their time.

### Questionnaires

Levels of anxiety symptoms, depression symptoms, rumination and worry were assessed by the following questionnaires, respectively: the Depression Anxiety Stress Scales (DASS-21^3^), the State–Trait Anxiety Inventory–Trait Version (STAI-T^27^), the Beck Depression Inventory–Second Edition (BDI–II^28^), the Ruminative Responses Scale (RRS^29^), and the Penn State Worry Questionnaire (PSWQ^30^); see “Supplementary Material” for psychometric evaluation and further elaboration for each questionnaire.

### Behavioral measurements

Readers are referred to the “Supplementary Material” section for further information about the stimuli validation and description of each task.

**Figure 1:**
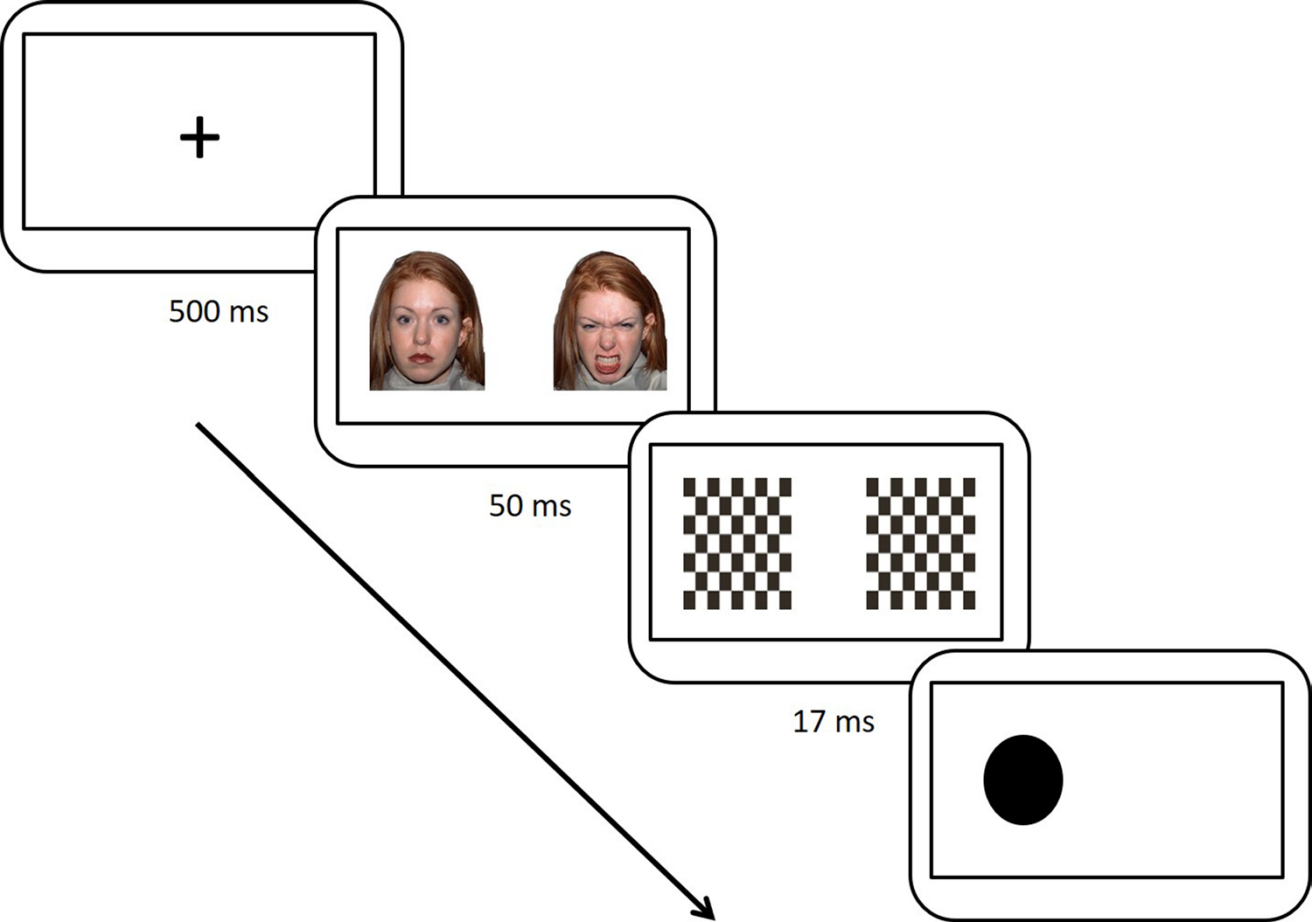
Emotional dot-probe procedure. Example of an incongruent trial from the sub-threshold presentation block. The figure shows a trial with neutral and angry faces. A target dot is presented at the location previously occupied by the neutral face. Reaction times for trials in which the target is presented in locations previously occupied by neutral faces and trials in which the target is presented in the previous location of emotional faces are compared. Therefore, they serve as measures of attention bias toward the emotional face cues.

Biases in spatial attention (abnormal orienting of attention to negative stimuli, either enhanced orienting or avoidance) was examined using an emotional dot-probe task (EDPT^31^; Fig. 1). Participants were requested to indicate the location of a probe appearing at a location previously occupied by either an emotional (happy/sad/angry) or neutral face. Attention bias was measured by reaction times (RTs). Faces were presented in either a sub-threshold manner (exposure duration of 50 ms) or a supra-threshold manner (1,000 ms), to examine automatic or elaborated processing biases, respectively.

**Figure 2:**
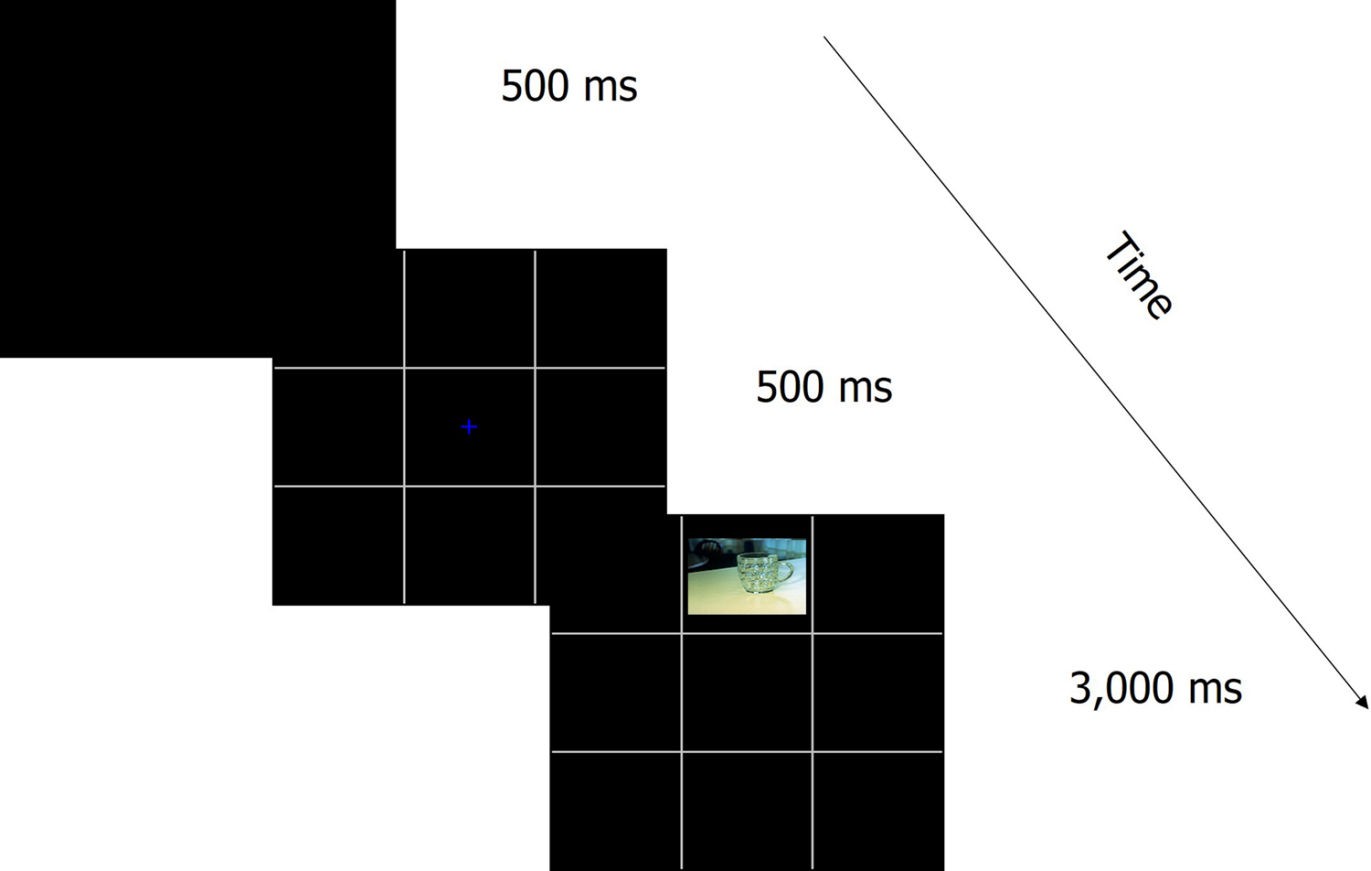
Focused attentional flanker task. Example of positive distracting pictures in an incongruent-location trial. The distracting pictures could be positive, negative or neutral. The central target picture can appear above or below the fixation point. Participants are asked to respond to the location of the target picture and ignore the distracting side pictures.

Biases in selective attention (the ability to ignore distracting emotional information) was assessed with a focused attention flanker task (FAFT^32^; Fig. 2). Participants were asked to determine the location of a neutral picture target and to ignore emotional (positive/negative valenced) or neutral flankers at the sides of the visual field. The flanking pictures appeared at congruent or incongruent locations (thus, distractors included spatial relevant-to-task and emotional not-relevant-to-task factors). Interference with attention was measured by RTs.

**Figure 3:**
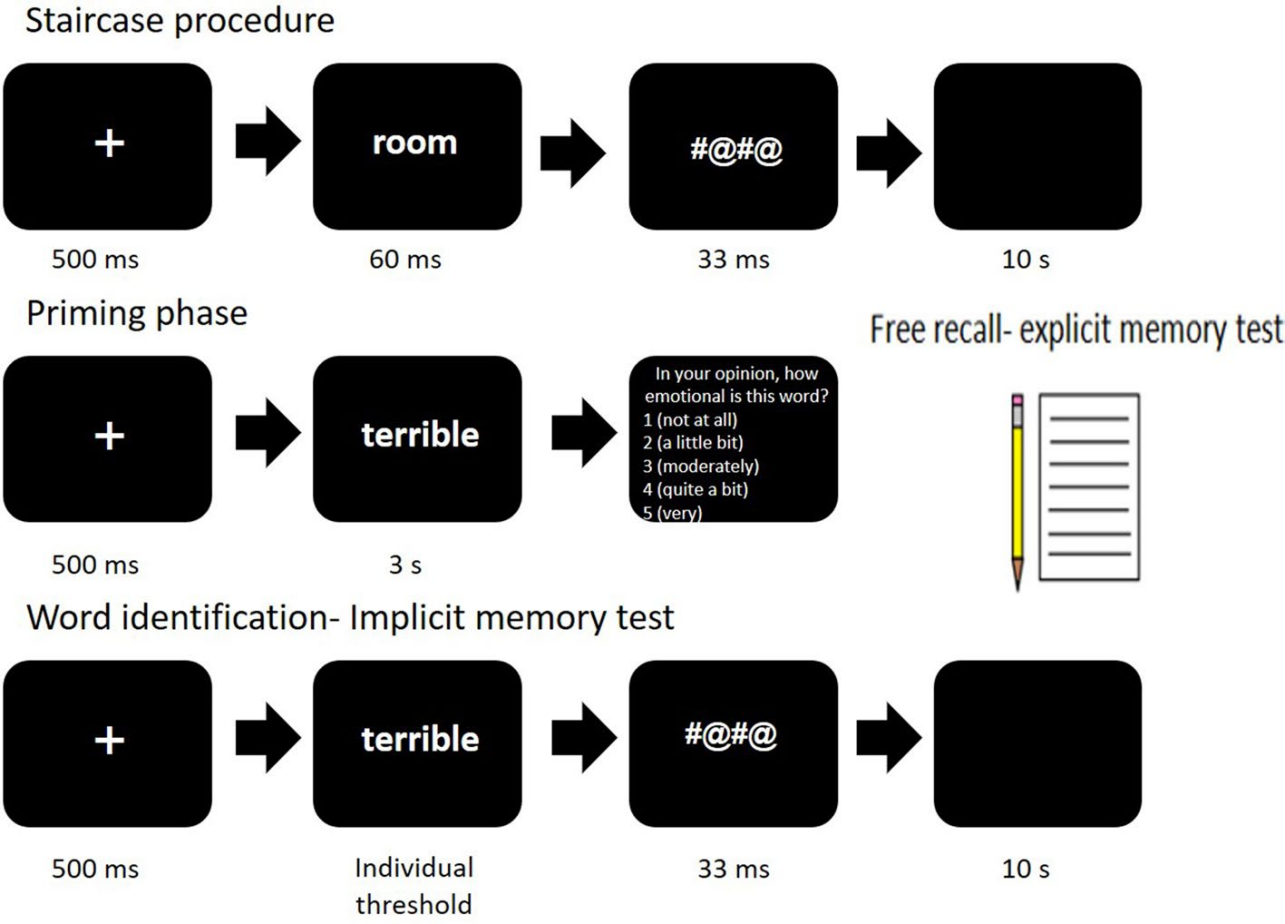
The word identification task. The task is based on an initial staircase procedure to set an individual threshold for word recognition, followed by priming, implicit and explicit memory tests. Memory for emotional versus neutral words is compared.

Biases in memory (abnormal memory of emotional versus neutral items, compared to healthy participants) were assessed using the word identification task (WIT^33^; Fig. 3). The WIT includes four stages. First, an individual threshold for word recognition is determined. Then, participants are asked to rate positive, negative and neutral words that are presented in a supra-threshold manner. After that, an implicit memory test is given in which words are presented in a sub-threshold manner. The final step includes an explicit memory recall test. The WIT examines implicit and explicit memory traces. Automatic bias was examined by an implicit memory test, in which participants were requested to read aloud sub-threshold emotional (negative/positive) or neutral words. Half of the test words were presented previously in the priming phase. Non-automatic memory bias was examined by a five-minute free recall test at the end of the task, measuring explicit memory of the words.

Biases in interpretation (interpretation by anxious or depressed participants compared to healthy participants of ambiguous situations as more negative) was examined using a modification of the word–sentence association paradigm (WSAP^34^). Ambiguous sentences (e.g., you hear a noise in the night) were followed by benign and negative interpretations (e.g., dog vs. burglar). Participants were requested to choose the more relevant word in their opinion (non-automatic interpretation) and RTs were measured for indication of automatic interpretation.

Expectancy biases (abnormal expectation of negative future events) was assessed using a future events task (FET^35^). Participants were requested to answer whether positive or negative events are likely to happen to them in the future (e.g., I will have a successful career, I will not be able to deal with responsibility), RTs were measured for indication of automatic expectancy. Certainty rating followed.

**Figure 4:**
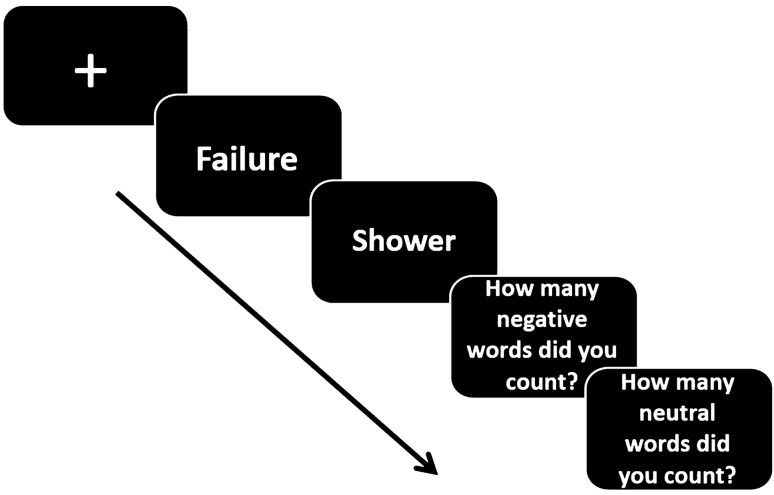
Internal switching task procedure. Example of the emotional version. Emotional and neutral words are presented sequentially at the center of the screen. Participants are asked to mentally count how many negative as well as neutral words appear. Once every few trials, they are asked to report the number of words in each mental count list.

Cognitive control was assessed by the internal switching task (IST^36^; Fig. 4). Participants were requested to keep a mental count of how many words they see from two different categories. The order of presentation results in switching and non-switching between category conditions. RT measurement of the counting itself indicates the cognitive cost of switching, which reflects the deficit in cognitive control. Each participant completed a neutral (noun and verb categories) and an emotional version (negative and neutral categories) version of the task.

### Procedure

Participants took part in the study during two sessions of approximately 1 h each, with an interval of 3–8 days between sessions. At the beginning of each session, one version of the IST was administered, to avoid the influence of fatigue on task performance. The order of the rest of the computational tasks and the two versions of the IST was counterbalanced between participants and between sessions, to avoid possible carryover or order effects. In addition, tasks with similar stimuli (i.e., words, sentences or pictures) were not administered consecutively (e.g., the WIT was not administered immediately after the IST). This method yielded 12 possible permutations for task order, and each participant was randomly assigned to one of them. To avoid a possible effect on task performance, participants completed the personality questionnaires at the end of the second session. At the end of the first session, participants received a document specifying professional mental health bodies and were encouraged to consult them if they were experiencing any distress. At the end of the second session, participants were debriefed and thanked for their participation.

### Data analysis

Readers are referred to the “Supplementary Material” for information about the data cleaning process.

The machine-learning algorithm, coded in MATLAB software, partitioned the entire dataset into training (80% of the data) and validation sets (20% of the data). This partitioning was done a hundred times, each time with randomly different participants from each group. Further, to account for the different group sizes, the number of participants selected from each group was equivalent to the smallest group in each model. This process of selection was done 10 times. In each selection, randomly different participants were chosen from each group according to the number set by the smallest one. For the symptomatic versus non-symptomatic model: 47 participants from each cluster (symptomatic groups versus control) were chosen for the training phase and 12 from each cluster for the validation phase. For the anxiety versus depression model: 16 participants were chosen from each group for the training phase and four from each group for the validation phase. This process of selection was done a 1,000 times (10 × 100). Therefore, the aggregated raw numbers of the training and validation groups are higher and include all participants, with different ones in each partitioning. The training data were used to construct a classifier that identifies patterns in the behavioral measures. These patterns correspond to the different symptomatic profiles as defined by the questionnaires. The classifier is essentially a function that receives the behavioral measures and outputs the symptomatic profile.

Once the classifier was constructed, the behavioral measures of the validation sets were processed by the classifier. The output for each participant was then compared with his or her true symptomatic profile. The level of agreement between the classifier decisions and the symptomatic profiles indicates the method’s performance level. The higher the success rate, the better the method.

Within this project, over 60 different behavioral measures were recorded for each participant. Some measures contributed to the classification of the various symptomatic profiles and some did not. To infer the importance of the various behavioral measures, a ‘leave-one-out’ analysis was used, where at each round one measure is omitted from the classification procedure. The success rate of each iteration, with one feature omitted, is then compared with the success rate of the classification with all measures. The lower the success rate with one measure omitted with respect to the classification success with all, the higher this specific measure’s importance. If the success rate with a specific measure omitted is higher than the success rate with all measures, then this measure interferes with the classification—that is, it has a negative contribution and should not be included in the classification process. The importance of the jth feature is computed in the following fashion: For the set of trees obtained in the training phase, the validation is run twice: once for the original dataset and once for the same dataset with the jth feature’s data permutated between patients. Then, the error difference between the two runs, for each tree, is computed, generating a differences series, one difference for each tree. The importance of the jth feature is the error series average divided by its standard deviation. Thus, after performing the ‘leave-one-out’ analysis, only measures that made a positive contribution were used.

Once the behavioral input measures were selected, the classification took place. A machine-learning analysis method based on a *random forest* algorithm^37^ was designed, so that the trees were sufficiently sensitive and could classify participants into the four study groups. A random forest consists of a set of decision trees, 1,000 in the current study, where each tree is a set of consecutive rules that divide the input data, so the resulting groups are more homogeneous with respect to the questionnaire labeling^38^. The process is repeated for the sub-groups, if those contain members having different questionnaire classifications. The process ends when all groups and sub-groups are homogenous. The creation of two sub-groups from a group, at each iteration, is done by dividing the group by applying thresholding on one of the input measures. In this way, measures that do not present clear separation of the data into homogenous sub-groups will not be used in the process. This mechanism, which is inherent in decision trees, serves as feature selection, helping to avoid over-fitting of the data. In the forest, each tree has its own set of rules, but all trees use the same input training data. The use of a random forest facilitates accurate classification in the presence of large variance of the measures within each group, because the forest allows for a large number of repetitions (trees) in the classification process. In this way, even subtle trends manifest themselves in the classification results^39^. Once all the trees are constructed, the classification decision is made by aggregating all the trees’ classification labels in a voting mechanism. Once classified, the trees’ classification labels are verified by applying these rules on the validation dataset. The rules, determined already in the training phase, are not changed in the validation stage. The outcome of the voting of the forest for each participant is then compared to its corresponding questionnaire labeling.

The training phase depends heavily on the selection of the training data. To avoid any selection bias, the entire forest classification process is repeated a thousand times, where at each run the training set is selected randomly and, therefore, each participant is selected randomly about 25 times. The standard deviation of the method’s accuracy over all iterations was very small, showing that the method is stable and the selection bias, if exists, is marginal.

The literature offers two common methodologies for assessing classification results: confusion or error matrices^40^ and McNemar’s test^41^. Both were used in this study. The former method is widely used in machine learning for assessing statistical supervised classification, as is the case in the analysis presented here (e.g., see Lugger and Yang^42^ for an example of classification of emotional features). In the confusion matrix, each matrix row represents the instances in a predicted class while each column represents the instances in an actual class (or vice versa). The correctly classified members of the dataset reside along the matrix’s diagonal. The algorithm’s accuracy is then measured by the trace of the matrix divided by its sum of all elements. McNemar’s test aims at evaluating paired dichotomous data^43^. Specifically, in this study, two classification algorithms’ results performed on the same dataset, against the true labels, were tested to detect which classification performs better and then whether the difference between the misclassification rates is statistically significant. In psychology, McNemar’s test was used recently for assessing computer analysis of psychiatric data^44,45^. In this study, we employ both methods to give the reader a comprehensive evaluation of the classification results.

The software package will be available on the authors’ website upon acceptance.

## Results

Symptomatic versus non-symptomatic model (HA + HD + HAD groups versus LAD group).

**Figure 5:**
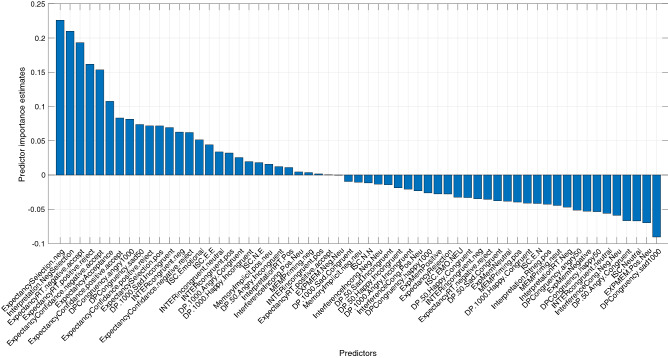
Marginal contribution of each behavioral measure. The normalized error difference between the classification of all the behavioral features, with and without each specific measure as indicated on the X axis, is shown. The larger the difference is, the larger the unique contribution of the specific behavioral measure. The jth feature importance is computed in the following fashion: For the set of trees obtained in the training phase, the validation is run twice—once for the original dataset and once for the same dataset with the jth feature’s data permutated between participants. Then, the error difference between the two runs, for each tree, is computed, generating a differences series, one difference for each tree. Accordingly, the jth feature importance is the error series average divided by its standard deviation. Measures that are located to the left of the zero-point showed a positive contribution to the model and were inserted into the bagged tree algorithm. Thus, after performing the ‘leave-one-out’ analysis, only measures that made a positive contribution were used. Out of 61 behavioral measures that were analyzed, 27 were found to contribute to the model prediction (18 did not). As seen on the left of the X axis, the measure contributing the most was the percentage of acceptance of negative sentences in the FET, which measures expectancy biases. In descending order, this was followed by the percentage of negative selection in the WSAP, which measures interpretation biases. The least contributing measure was an EDPT index of sad facial expressions, presented for 1,000 ms. This measure was, therefore, not inserted into the final algorithm.

The *leave-one-out measure analysis* revealed the marginal contribution of each behavioral measure drawn from the input. Figure 5 shows the normalized error differences between the classification of all the behavioral measures, with and without the specific measure. The larger the difference is, the larger the unique contribution of the specific behavioral measure. Measures that are located to the left of the zero-point indicate a positive contribution to the model and were inserted into the bagged tree algorithm. The measure that was found to contribute the most to the classification was percentage of negative selection in the FET (which measures expectancy biases), followed by the percentage of negative selection in the WSAP (which measures interpretation biases).

The *bagged decision tree classification* algorithm revealed 70.78% specificity, and 71.44% sensitivity. Table 2 shows the classification accuracy of the participants in each cluster.

**Table 2:** Classification accuracy.

True group		
Cluster	HA + HD + HAD	LAD
**Classification prediction**		
HA + HD + HAD	**0.71**	0.29
LAD	0.30	**0.70**

Accuracy rates represent the classification prediction of each participant, which was based on his or her behavioral measures, as compared to the participant’s true group membership, which was based on his or her symptomatic profile.

McNemar’s test was better at distinguishing between the groups by classifier compared to random classification (χ^2^ (1) = 7.17, p = 0.00054353).

To test the strength of the algorithm in differentiating solely between depression vs. anxiety symptomatic profiles, a second two-group model was introduced into the classification scheme.

### Two-group model results (HA vs. HD)

**Figure 6:**
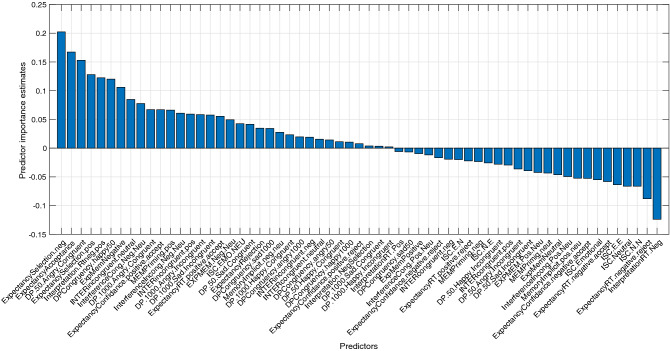
Marginal contribution of each behavioral measure. The normalized error difference between the classification of all the behavioral features, with and without the specific measure on the X axis, is shown. The larger the difference is, the larger the unique contribution. Measures that are located to the left of the zero-point demonstrated a positive contribution to the model, and were inserted into the bagged tree algorithm. Out of 61 behavioral measures that were analyzed, 34 were found to contribute to the prediction success, while 27 were not. The most-contributing measure was percentage of negative selection in the FET, which measures expectancy biases, followed by index of RT differences between positive and negative selections in the same task. The least-contributing measure that was not inserted into the algorithm was the mean RT for negative selection in the WSAP.

The *leave-one-out* analysis revealed the marginal contribution of each behavioral measure drawn from the input, as shown in Fig. 6. The measure that was found to contribute the most to the classification was percentage of negative selection in the FET, which measures expectancy biases, followed by index of RT differences between positive and negative selections in the same task.

The *bagged decision tree classification* algorithm revealed a 68.07% success rate in predicting participants in the HD group, and a 74.18% success rate in predicting participants in the HA group. Table 3 shows the accuracy of the classification of participants into each group.

**Table 3:** Accuracy rates represent the classification prediction of each participant, which was based on his or her behavioral measures, as compared to the true group membership, which was based on his or her symptomatic profile.

True group		
Group	HA	HD
**Classification prediction**		
HA	**0.74**	0.26
HD	0.32	**0.68**

McNemar’s test was better at distinguishing between the groups by classifier compared to random classification (χ^2^(1) = 4.07, p = 0.03039917).

## Discussion

The current study sought to differentiate between subclinical levels of anxiety and depression by detecting if a unique pattern of biased reactions to emotional stimuli exists for each disorder, based on participants’ aggregated performance in several behavioral tasks. Attention, memory, interpretation and expectancy biases were examined, as well as cognitive control ability. Data were analyzed by machine-learning tools that predicted the group membership of each participant according to his or her performance on the tasks. The analysis reached 71% and 70% sensitivity and specificity, respectively, and 68% and 74% classification accuracy of the HD and HA groups, respectively, in a two-group model. These classification accuracies were all above chance level. These results show the importance of combining behavioral measurements and machine-learning methods in the field of psychiatric diagnostics. Specifically, we suggest that machine-learning analysis tools may help reach higher confidence during the diagnostic procedure. In contrast to current diagnosis, which is based on self-reported symptoms, the cognitive tasks examine behavioral measures such as subtle differences in reaction time, which are less prone to self-report biases. Furthermore, the battery provides a comprehensive picture of the characteristic cognitive patterns, which can serve for future development of individually-tailored treatments. In the following, we elaborate and discuss the possible meaning and implications of the findings, as well as the novel implementation of machine-learning analysis tools in the context of cognitive biases in psychiatry. Further, we present the limitations of the current study and offer future research directions.

### The importance of multiple measure-based analysis

Notably, measures from *all* the tasks included in the test battery contributed to the categorization prediction. Thus, the ability to predict group classification would have been lower without the representation of these various functions. This finding demonstrates the existence of deficiencies in different cognitive mechanisms among depressed and anxious individuals. Such deficiencies are reflected in an increase in explained variance when inserting measures from several cognitive categories. Furthermore, as seen in Figs. 5 and 6, both overlap and variance exist between the behavioral measures that contributed to the prediction as well as in the degree of their contribution in each model. Therefore, differences between anxiety and depression cannot be based on a single cognitive function, but must be looked for in a combination of performance patterns in different functions. Machine-learning analyses enable us to connect the data from different bias categories, to comprise a united pattern characterizing each group.

### The contribution of automatic processing to the prediction

The results of the current study support the notion that different levels of automaticity of processing differentiate between anxious and depressed individuals. Measurements of different levels of automaticity in the same bias category were each found to contribute to the prediction (e.g., both implicit and explicit memory).

The behavioral test battery was developed in a manner that allowed for collection of data on both automatic and more elaborated reactions. The literature suggests that biases toward emotional stimuli are mostly automatic among anxious individuals, but not so among depressed individuals, whose biases are typically not automatic. Rather, their occurrence requires longer processing and further elaboration of the stimuli (^18,19^, see^46–48^ for various definitions of automatic processing). Several reviews have summarized findings regarding the differences in cognitive processing between anxiety and depression. They found that the automaticity levels contribute to the differentiation to some extent^14^.

### Bagged decision tree classification results

#### Differentiation between high and low levels of anxiety and depression symptoms

The results of the bagged decision tree classification, in the symptomatic vs. non-symptomatic models, point to the algorithm’s high classification sensitivity as well as to the strong impact of depression and anxiety symptoms on cognitive mechanisms. Based on participants’ cognitive biases toward emotional stimuli, the algorithm displayed an excellent ability to differentiate between individuals with low levels of anxiety and depression symptoms (the LAD group) and individuals who exhibit high levels of anxiety and/or depression (the HA, HD, and HAD groups). From a clinical point of view, these results show the impact of subclinical levels of depression and anxiety symptoms on the everyday life of individuals suffering from anxiety or depression characteristics. These individuals are likely to cope with the consequences of their biased processing patterns, and may not seek psychological or psychiatric treatment since they are not clinically diagnosed.

#### Differentiation between depression and anxiety symptoms

The results obtained from the two-group model are of diagnostic importance. The battery of the behavioral tests that was developed for the current study, along with the machine-learning algorithm, were able to classify participants to the HD and HA groups 18–24% more accurately than chance level, based solely on their behavioral reactions. Even though the population in the current study is not clinically diagnosed, these results lay the ground for future studies. Currently, psychiatrists worldwide rely exclusively on self-reported symptoms to make a differential diagnosis of anxiety and depression^49^. To reach higher confidence in the diagnosis, additional aids alongside symptom-based diagnostic tools should be developed.

#### Limitations and future directions

The main limitation of the current study is the rather small sample, when considering each group separately. This drawback is especially relevant as the findings were not validated by an external dataset but by using an internal validation procedure on a random 20% of the data in each iteration (see, for example^50^ for evidence that machine-learning studies with small samples that were not validated in external datasets resulted in decreased prediction accuracy). Future studies are planned to validate the findings in external datasets. Recruiting participants with high (albeit subclinical) levels of anxiety and/or depression symptoms is a difficult task, for two reasons: First, such participants refrain from participating in experiments compared to subjects with low anxiety or depression levels. Second, the occurrence of high levels of anxiety and depression symptoms in a normal population is rather low (e.g., 5.2% and 5.8% of participants were found with severe or extremely severe levels of anxiety and/or depression symptoms, respectively, in a study among a healthy population^51^). Therefore, in our study, only 59 participants with high levels of anxiety and/or depression were recruited, out of more than 400 volunteers that filled out the screening questionnaire. These 59 participants were then divided into three relatively small groups. Further, a minor percentage of the data was missing due to technical errors (see “Supplementary Material”). Data were missing, however, occurred in a very small proportion of the overall data. Furthermore, missing data were evenly distributed between the groups. Therefore, it is unlikely that this missing data had a significant effect on the results.

Another limitation is that the study was performed in a subclinical sample. Therefore, conclusions regarding clinical diagnosis and treatment should be drawn with caution. The findings should be interpreted as a proof-of-concept for the combination of the cognitive battery with machine-learning classification analysis. Considering the above, future studies may examine the tools developed here in clinical populations. Such studies will determine the efficacy of the current methodology as a decision support system during psychiatric diagnosis.

Similar to other studies using machine-learning classification, another limitation lies in the fact that the cognitive battery is validated using questionnaires, which are inherently prone to self-report biases and are not 100% accurate. This bias, however, is mitigated by the fact that the participant’s classification is not evaluated solely on their answer to the questionnaires, but also on how it compares to all previous questionnaires completed by all participants and scored thus far.

In a future study and following additional validation in clinical samples that are diagnosed based on a comprehensive interview with a psychiatrist, the cognitive tasks would be evaluated against a questionnaire as well as a clinician interview. This would increase the robustness of the method.

The conclusions drawn from exploring the specific reaction patterns presented by individuals suffering from each disorder may in the future produce clinical benefits. They could be of great help, for instance, if implemented in cognitive training such as *cognitive bias modification* (CBM). CBM seeks to modify cognitive processes into being more adaptive to daily life^7^, and was found to improve psychopathological symptoms^52^. Shani et al.^53^, reviewing the cognitive training literature, surmised that its efficacy is highly affected by the intervention selection—a central approach in personalized medicine^54^. Intervention selection aims to optimize intervention efficacy by identifying the most beneficial type of intervention for a given individual. Machine-learning approaches may be highly suitable for such identification. They enable the selection of the items that contribute the most to a treatment, without relying on a specific theory. In a recent study^55^, implementation of the variables that were found to increase treatment efficacy by machine-learning algorithms indeed resulted in improved treatment.

In sum, employing a battery of cognitive–behavioral tasks analyzed by machine-learning algorithms developed for the current study, we show the importance of advanced analysis methods based on multiple measures to better characterize subclinical psychiatric disorders. The algorithm developed in the current study differentiated between participants with high levels of anxiety and/or depression symptoms and participants with low symptoms levels, as well as identified specific reaction patterns exhibited by depressed and anxious individuals. These observations pave the way for studies aimed at finding classes that stem from cognitive–behavioral data that may indicate these (and possibly other) disorders. Further, these findings lay the foundation for the development of more specific and refined diagnosis and clinical treatments, to be supported by future studies that will further expand the growing knowledge in the field of cognitive biases among psychopathological populations.

## Supplementary information

Supplementary Information 1.

Supplementary Information 2.
