# Supplementary material for: Using machine learning-based analysis for behavioral differentiation between anxiety and depression
Source: Sci Rep. 2020 Oct 2;10:16381. doi: 10.1038/s41598-020-72289-9 (PMC7532220; doi:10.1038/s41598-020-72289-9)
Supplement: Supplementary file 1 — Supplementary Information 1. [file 41598_2020_72289_MOESM1_ESM.docx]

Using machine learning-based analysis for behavioral differentiation between anxiety and depression

Authors: Thalia Richter^1*^, Barak Fishbain^2^, Andrey Markus^1^, Gal Richter-Levin^1^, Hadas Okon-Singer^1^

Institutional affiliation-^1^Department of Psychology, University of Haifa, Mount Carmel Haifa, Israel; ^2^Faculty of Civil and Environmental Engineering, Technion – Israel Institute of Technoloyg, Haifa, Israel.

Please send correspondence to Thalia Richter, Department of Psychology, University of Haifa, Mount Carmel, Haifa 3498838, Israel; thalia.richter173@gmail.com

**Supplementary material**

*Missing* *data*

Because of a technical error, the demographic information of five participants is partially missing, thus the data regarding age, sex and education years are based on 1–5 participants less than the total number of participants in each of these categories. In addition, the scores of four participants on the DASS-21 questionnaire are missing. These participants were assigned to the experiment groups (two for HA group, two for HAD group) based on their scores on STAI-T and BDI-II, according to the recommended cut-offs scores. These questionnaires were found to be highly correlated with the DASS-21 [56–57]. Thus, it is reasonable to assume that a score above the cut-off level for high symptoms in the BDI-II and/or STAI-T would have been accepted in the DASS-21 as well. Additionally, the data of the explicit memory test are missing for four other participants (three from the LAD group, of which one also did not perform the explicit memory test, and one from the HAD group), and from the WSAP, for one participant. Therefore, the results obtained from these two tasks are based on 1–4 participants less than the total number of participants. Finally, six participants did not answer one question in one of the questionnaires (without a trend of a specific questionnaire). As recommended in these questionnaires’ manuals, the average score of the questionnaire without the missing question was inserted as their answer to the missing question.

*Psychometric evaluation of questionnaires*

(A) The Depression Anxiety Stress Scales [DASS-21; 3] is a set of three self-report scales designed to measure the emotional states of depression, anxiety and stress. Each item includes a statement about the self, rated on a four-point Likert scale. Cronbach’s alpha for internal consistency is higher than .82 for all the questionnaires’ scales [58]. Convergent and discriminant validity with other measures of the traits are adequate as well.

(B) The State–Trait Anxiety Inventory–Trait [STAI-T; 27] consists of 20 self-report statements evaluating the general tendency to experience anxiety and sensitivity to anxiety-evoking situations, rated on a four-point Likert scale. Cronbach’s alpha for internal consistency, as reported in Barnes, Harp, and Jung [59], is .89. Spielberger [60] showed good convergent and discriminant validity with other measures of trait anxiety.

(C) The Beck Depression Inventory–Second Edition [BDI-II; 28] assesses the cognitive, affective, behavioral and somatic aspects of depression. The instrument contains 21 items, each referring to a specific symptom of depression in accordance with DSM-IV criteria (e.g., changes in sleep patterns, loss of energy, feelings of worthlessness). Cronbach’s alpha for internal consistency is .91, and convergent and discriminant validity were found adequate by Beck, Steer, and Brown [61].

(D) The Ruminative Responses Scale [RRS; 29] consists of 22 items assessing behavioral or mental responses to a dysphoric mood. The scale has been found to correlate strongly and positively with depression symptoms and mixed anxiety/depressive symptoms [62], with or without the items that relate directly to depression [63]. Cronbach’s alpha for internal consistency, as found in Roelofs, Muris, Huibers, Peeters, and Arntz, [64] is .90, and convergent and discriminant validity were found to be adequate as well.

(E) The Penn State Worry Questionnaire [PSWQ; 30] is a 16-item questionnaire designed to glean information about the frequency and intensity of the act of worrying in general, without any content specificity. Scores on the PSWQ correlate highly with symptoms of anxiety [65]. PSWQ has high internal consistency (α= 0.95) and test-retest reliability (α=0.92) as well as satisfactory convergent validity [30].

*Stimulus validation process*

A pool of 440 words was created for the current study by assembling and validating words from various scientific resources. All neutral words and positive valence words and a small portion of the negative valence words were taken from a set of Hebrew words validated in Israel by 21 participants [66]. These participants rated the valence of 800 words by answering the following question on a scale from 1–9: “When you refer to the meaning of the word, in your opinion how positive, negative or neutral is it?” In the current study, the valence for the positive words ranges from 1.77–2.94, for the neutral words from 3.36–5.57 and for the negative words from 7.24–8.57. Additional negative words used were based on previous studies examining biases among anxious and/or depressed populations [31, 33, 67–70]. In all these studies, the negative words were separated into anxiety-related words and depression-related words. Many words, however, yielded mixed results (e.g., categorized as anxiety-related in one study and as depression-related in another). To avoid this ambiguity, in the current study we validated the Hebrew words by asking five independent raters to rate each negative valence words on two scales. The raters were asked to indicate to what extent each word is related to depression and then to anxiety on a five-point Likert scale ranging from “very little” to “very much”. After the results were analyzed, attempts to replicate previously used cut-off scores for categorization into two different groups [e.g., see 33] failed to yield a significant number of words fitting the terms. Thus, all words that received a score of 3 or above on both scales were chosen to be used in the current study and defined as negative words, without specific condition categorization.

In most of the above-mentioned studies, results showing biases among the anxiety/depression group were usually confined only to the relevant words-category for the specific group. This pattern of findings limits the conclusion that can be made regarding the abnormal reactions to emotional stimuli among these populations. In other words, instead of making inferences about the reaction to negative words, one can only conjecture about the reaction to specific anxiety/depression-related words.

This unspecific-condition valence attribute is in line with recent theories in the field of emotions, suggesting that an emotional stimulus will not activate a specific brain area assigned to a discrete emotion; rather, it will trigger a network of brain areas operating on a broad range of emotions [71]. Thus, it is possible, and even more likely, that the individual will experience several psychological constructs simultaneously, as was also suggested by Kron, Goldstein, Lee, Gardhouse, and Anderson [72].

The Word Identification Task (WIT) and the Internal Switching Task (IST) yielded no significant differences (all p values above .23) between the words from the different categories in terms of word length and frequency of appearance in Hebrew [data derived from The Word-Frequency Database for Printed Hebrew; 73].

The ambiguous sentences and negative interpretations used in the Word Sentence Association Paradigm (WSAP) underwent a validation process similar to the one described above in regard to our pool of words. Here, sentences were taken from three different studies: Beard and Amir [34], Hindash and Amir [74], and Ogniewicz et al. [75]. In these studies, the method was identical but the sentences differed for participants with high depression symptoms and participants with high anxiety symptoms in that they were believed to describe situations relevant to each specific condition separately. To validate this categorization, five independent raters rated each sentence with its negative interpretation as described above. The same cut-offs were applied and again yielded an insignificant number of sentences in every group. Therefore, all interpretations with a score of 3 or above on both scales were chosen to be used in the interpretation task without specific condition categorization.

All experiments were programmed with E-prime software (version 2.0) and shown on a PC computer with a refresh rate of 100 Hz.

*Elaboration and technical description of the behavioral tasks*

**Emotional dot-probe task** [based on 31]: The task includes 365 pictures of average human facial expressions modeled by 93 amateur actors (49 male and 44 female) expressing happy, threatening, sad and neutral emotions. Facial expressions are derived from two validated sets: The NimStim Face Stimulus Set [76], and the Karolinska Directed Emotional Faces [KDEF;77], both used frequently in previous studies of the emotional dot-probe task [e.g., 78–80]. The two sets were developed under similar conditions in terms of the actor’s clothes, the actor’s distance from the camera and the requirements concerning appearance, picture quality and lighting. Hence, they were merged into one larger set with no apparent differences.

The task is composed of two blocks. The first is a sub-threshold block and the second a supra-threshold block. Each block comprises 180 trials, with each emotional facial expression presented for an equal amount of time in random order. Every trial starts with a fixation cross in the middle of the screen presented for 500 ms. A black square frame surrounds the inner square of the screen to focus the participant’s attention on the center. The fixation cross remains on the screen while two facial expressions appear to its left and its right. The center of each picture is located at a 6º angle from the center. The pictures are 8.38 cm long by 6 cm wide, with participants seated 80 cm from the screen, thus enabling them to capture the entire scene in their visual field, with no need for saccades [81]. In every trial, a neutral facial expression appears alongside an emotional expression modeled by the same model. In the supra-threshold block, the expressions appear for 1000 ms, followed immediately by a round probe placed in one of the locations of the pictures. In half of the trials, the probe is placed in the emotional expression location (congruent) and in the other half, in the location of the neutral expression (incongruent). The locations of the neutral and emotional facial expressions are counterbalanced between trials to prevent the effects of side bias. In the sub-threshold block, the pictures appear for 50 ms and are then immediately followed by a mask of checkerboards covering their exact location. The mask appears for 17 ms, and then the probe appears as in the supra-threshold block. Participants are instructed to press on the left or right arrow key on the keyboard according to the probe location, as quickly as possible but not at the expense of accuracy. Each block starts with four practice trials. After every 60 trials, participants are given a break.

**Focused attentional flanker task** [adopted from 32]: This task examines interference to attention possibly caused by emotional aspects of distractors, which are irrelevant to task performance. The task includes 20 practice trials, followed by four blocks of 96 trials, separated by a break offered to the participants. Each trial starts with a black screen for 150 ms, followed by a black rectangle divided into nine smaller equal-sized rectangles. Each small rectangle is 8 cm (width) X 7 cm (height). The array of rectangles creates a 3 (columns) X 3 (rows) grid. In each trial, a neutral target picture and two flanking identical emotional pictures appear inside the grid. The flankers always appear in the left and right columns. The neutral target appears randomly above or below a central fixation cross (inside the focus of attention) that appears for 150 ms in the middle column. The flankers can appear above, below or at the level of fixation. Hence, the location of the flankers is congruent, neutral or incongruent with the location of the target. The valence of the flanker pictures changes randomly between trials to positive, neutral, or negative. The target always has a neutral valence. Thus, flanker location should affect performance (relevant to task demands), but an effect of flanker valence will reflect interference to attention since it is irrelevant to the task.

The 90 pictures for each valence in the task are taken from the International Affective Picture System [IAPS; 82]. Mean valence values are 2.1, 5.1 and 7.5 for negative, neutral and positive pictures, respectively, on a scale of 1–9, and mean arousal values are 6.1, 3.3 and 4.9 on a similar scale [82–84]. Participants are instructed to indicate whether the target picture is located above or below the central fixation cross by pressing one of two keys: The P key for above and the Q key for below. They are asked specifically to direct their attention to the fixation cross and disregard other stimuli. The target and the flankers remain in view until the participant presses a key, but for no more than 3000 ms. Participants are asked to respond as quickly and as accurately as possible. The intertrial interval (ITI) is 1500 ms, measured from the onset of the participant’s response.

**Word Identification Task (WIT)** [modified from 33]: The task, which examines implicit and explicit memory, is divided into four phases. The aim of the first phase is to find each individual’s visual perception threshold so as to present the words in the third phase at sub-threshold duration. This is accomplished using a staircase procedure. Staircases usually begin with a high intensity stimulus that is easy to detect. The intensity is then reduced until the observer makes a mistake, at which point the staircase “reverses” and the intensity is increased until the observer responds correctly, triggering another reversal. The values for the last of these “reversals” are then averaged [85]. The current study employed a “1 up, 2 down” rule for correct answers and mistakes [86], in which every two consecutive correct answers at a certain exposure duration resulted in a reduction in duration, and each mistake resulted in returning to the former longer duration. A reversal is defined as an upward or downward reversal of the duration’s trend. Each trial begins by showing a fixation cross for 500 ms, followed by presentation of the target word for 33 ms. The target word is masked by a string of symbols matched in length to the letters. The screen is then cleared until the participant responds or until a maximum of 10 s elapses. After an intertrial interval (ITI) of 200 ms, the fixation cross appears again to prepare the participant for the next word. Participants are informed about the brief exposure of the stimuli, the presence of the mask, and the fixation cross at the center of the screen. In addition, participants are encouraged to guess all the words presented even if they are not sure or do not believe they actually saw a word, since it does not matter if their answers are right or wrong. Every target word is displayed on a second screen visible only to the experimenter, who codes the responses as correct or incorrect to set the next exposure duration. The initial exposure duration is set for 120 ms. The first reduction is of 20 ms and, after four reversals, it is shortened to 10 ms for higher detection sensitivity. After 80 trials or 14 reversals, the procedure stops. Each participant’s individual threshold is determined as the mean duration exposure for the last five reversals. All target words for the first phase are presented randomly, have neutral valences and are used solely during this phase.

In the second phase, 60 words (20 from each category: positive valence, negative valence and neutral) are presented in random order as a priming list. Participants are asked to pay attention to the words appearing one at a time for 3 s at the center of the screen and to rate them for their emotionality on a five-point Likert scale ranging from 1 (not at all) to 5 (very). The numbers appear after each word, and the rating scale remains on the screen until the participant presses the selected key on the keyboard. The next word then appears, preceded by a fixation cross shown for 500 ms. The aim of showing the priming list is for participants to implicitly remember it during the third phase and explicitly remember it during the fourth phase. To this end, the display time is relatively long and participants are asked to refer to the meaning of every word by rating its emotionality, thus facilitating a deeper level of encoding [87].

The third phase constitutes the implicit memory test. In this phase, the words are presented for a sub-threshold duration, and half of them are prime words from the previous phase. One-hundred and twenty words, 40 from each category, half already seen in the second phase and half new, are presented in the same manner as in the first phase of the experiment, except that the exposure duration is constant and adjusted according to each participant’s reading threshold detected in the first phase. To avoid a facilitating practice effect that might lower the individual threshold, the exposure duration for each participant is reduced by 30%, thus preventing a ceiling effect [33]. Six neutral words (chosen from the 80 neutral words shown during the first phase) are used as practice words.

After the end of the third phase, the explicit memory free-recall test is conducted. Only words from the priming list are analyzed, but participants are asked to write down all the words they can remember in five minutes to avoid false negatives (words not reported because falsely not attributed to the rating task).

The test is conducted after the implicit memory test because it is not clear how transient the phenomenon of priming may be in word identification [88]. The identification of words may remind participants of otherwise irretrievable words, though, on the other hand, recalled words may facilitate their identification. Moreover, immediate free recall would produce a recency effect, which is avoided by the randomized presentation of words during the third phase [33].

Given that some features of the task were modified for the current study, resemblance in performance to the original study [33] was examined to verify paradigm consistency. As expected, overall, more primed words were implicitly remembered than non-primed words. Further, means of remembering novel words were similar between the studies – 38% of novel words in both studies, showing that the paradigms of individual threshold identification worked in a similar manner.

**Word Sentence Association Paradigm (WSAP):** This task is a modification of the Word Sentence Association Paradigm developed by Beard and Amir [34] with some elements taken from Everaert, Duyck, and Koster [89]. Thus, the task comprises two prevalent methods of interpretation bias research among anxiety and depression subclinical populations. Thirty ambiguous sentences are randomly assigned to each participant from a pool of 127 self-relevant ambiguous sentences describing situations from different life domains (e.g., work, social life, family, romantic relationships, finance, academia, physical concerns) to achieve high generalizability [74]. Each sentence is presented with negative and benign associative words. The benign words are a mixture of related positive and neutral words. The two words relate to the ambiguous sentence and can interpret the situation described as negative or benign. Participants are asked to choose which word in their opinion is more related to the sentence. RTs are measured to examine the automatic bias of interpretation [34]. Participants are instructed to try to imagine themselves in the described situations. To facilitate the process, different sets of sentences are given to men and women using the appropriate gender form in Hebrew. Each trial begins with a fixation cross appearing for 500 ms, followed by a sentence presented for 3 s. After that, participants see a sentence asking them to choose which word is more related to the former sentence. The two possible choices appear on the left and right sides of the screen, and participants indicate their choice by pressing the left or right arrow key on the keyboard. Four sentences are given at the beginning of the task as practice trials.

Given that some features of the task were modified for the current study, resemblance in performance to the original study [34] was examined to verify paradigm consistency. It is challenging to compare between the two studies as their structure is different (rejection/acceptance of one valenced word in the original study vs. choice between two valenced words in the current study). Nevertheless, similar results in the overall aim of the task itself were found. In both studies, the symptomatic groups presented more bias toward threat and away from benign interpretations than the control group. In both studies, this trend manifested itself both in RTs and choice of negative interpretations (specific means were not provided in the original study).

**Future Events Task (FET)** [adopted from 35]: A few essential changes were made in order to measure implicit expectations about the future in addition to explicit expectations. Thirty-four sentences describing a future non-specific situation involving the self are presented one at a time in random order. Seventeen sentences describe positive situations (“I will have a successful career”; “I will have lots of good times with friends”), and 17 sentences describe negative situations (“I will not be able to cope with responsibilities”; “My family will disapprove of my life choices”). All the sentences are adapted from previous studies in which their valence was validated [e.g. 90–91]. The sentences were chosen based on their face validity – with ratings of moderate likelihood to occur to the average person [35]. Each sentence is shown for 3000 ms, preceded by a fixation cross shown for 500 ms and accompanied by a question: “Is this likely to happen to you at some time in the future?” The words “yes” and “no” appear on the right and left sides of the screen below the question. Participants are instructed to imagine themselves in the described situations and respond according to their belief about the likelihood this situation will occur in their future by pressing the right arrow for “yes” and the left arrow for “no”. This response indicates the participant’s explicit expectation. RT is measured to assess participants’ implicit expectation about their future. This is in accordance with previous evidence that response latencies can reflect implicit attitudes towards a given concept, with longer RTs correlated with implicit difficulty in accepting or rejecting [92–93]. After pressing the key, the participants are asked to indicate how certain they are of their response on a five-point Likert scale ranging from “not at all certain” to “very certain” by pressing the corresponding number on the keyboard. After that, the next sentence appears. At the beginning of the task, three neutral practice sentences are presented in the above-mentioned manner.

**Internal Switching Task (IST)** [modified from 36]: The IST is an indirect measure of cognitive control. The task is divided into two separate versions: neutral and emotional. The neutral version provides a “pure” measurement of shifting, updating and inhibiting, and the emotional version provides a measurement of the ability to control these functions while being exposed to stimuli evoking negative feelings. In the neutral version, 24 neutral verbs (looked, ringing) and 24 neutral nouns (nose, paper) are presented successively in random order. Participants are asked to mentally count, without any external aids, how many verbs and how many nouns they see and to update their count with every word. Each block of trials starts with a fixation cross for 500 ms, and then a noun or a verb appear in the middle of the screen until the participant presses the space button. Participants are instructed to press the space bar immediately when they finish updating their count. Hence, the RT for each word reflects the time needed for the mental action of the count and the cognitive functions required for it. After the participant presses the space bar, the next word appears. Each block contains 9, 13 or 17 words. The number of words varies across blocks to ensure that individuals cannot infer the number of words in the categories by means of subtraction. At the end of each block, participants are asked to report the number of words they mentally counted in each category, using the keyboard number pad. The order of the questions is counterbalanced to prevent pre-planning. Participants complete three practice blocks followed by 12 experimental blocks. The emotional version is identical to the neutral version, with one exception. It includes 24 negative valence words (failure, betrayal) and 24 neutral words (soup, shower), and participants are asked to count by these categories. All of the negative valence words are related to themes of fear of failure and relationships, themes that students, the majority of the participants in the current experiment, worry about the most [36, 94]. Therefore, they are likely to evoke strong reactions [95] as well as be harder to disengage from, when switching the internal count to the second category [36].

Given that some features of the task were modified for the current study, resemblance in performance to the original study [36] was examined to verify paradigm consistency. As for accuracy rates, the original study reported 83% for accurate counting and for +-1 away from correct response, while in the current study accuracy rates ranged between 68%–93% for +-1 away from correct response and/or response switched between categories. Further, in both studies, RTs for switch sequences were slower than for non-switch sequences (switch means – 1589 ms in the original study, 1758 ms in the current study, non-switch means – 1275 ms in the original study, 1403 ms in the current study), resulting in a similar average RT “cost” of 314 ms in the original study vs. 355 ms in the current study. These similarities demonstrate that both paradigms measured cognitive control deficits in an analogous manner.

*Data* *cleaning*

For all tasks, trials in which the RT measurement was above or below 2.5 standard deviations from the mean RT of the participant in that specific task were considered outliers and were eliminated from the data. For tasks in which performance accuracy was relevant (EDPT, FAFT), trials in which the response was wrong were also screened from the data. After these procedures, less than 3% of the total trials of each task, were screened.
