# Supplementary material for: Using machine learning-based analysis for behavioral differentiation between anxiety and depression
Source: Sci Rep. 2020 Oct 2;10:16381. doi: 10.1038/s41598-020-72289-9 (PMC7532220; doi:10.1038/s41598-020-72289-9)
Supplement: Supplementary file 2 — Supplementary Information 2. [file 41598_2020_72289_MOESM2_ESM.docx]

Supplementary material references

56. Lovibond, P. F., & Lovibond, S. H. The structure of negative emotional states: Comparison of the depression anxiety stress scales (DASS) with the beck depression and anxiety inventories. *Behav. Res. Ther.* **33**, 335-343 (1995).

57. McDowell, I. *Measuring health: A guide to rating scales and questionnaires.* (Oxford University Press, 2006).

58. Henry, J. D., & Crawford, J. R. The short‐form version of the depression anxiety stress scales (DASS‐21): Construct validity and normative data in a large non‐clinical sample. *Br. J. Clin. Psychol.* **44**, 227-239 (2005).

59. Barnes, L. L., Harp, D., & Jung, W. S. Reliability generalization of scores on the Spielberger state-trait anxiety inventory. *Educ. Psychol. Meas.* **62**, 603-618 (2002).

60. Spielberger, C.D., & Gorsuch, R.L. *Manual for the State-Trait Anxiety Inventory*. (Consulting Psychologists Press, 1983).

61. Beck, A. T., Steer, R. A., & Brown, G. K. *Manual for the Beck depression inventory-II*. (Psychological Corporation, 1996).

62. Nolen-Hoeksema, S. The role of rumination in depressive disorders and mixed anxiety/depressive symptoms. *J. Abnorm. Psychol.* **109**, 504; [10.1037/0021843X.109.3.504](https://doi.org/10.1037/0021-843X.109.3.504) (2000).

63. Treynor, W., Gonzalez, R., & Nolen-Hoeksema, S. Rumination reconsidered: A psychometric analysis. *Cognit. Ther. Res.* **27**, 247-259 (2003).

64. Roelofs, J., Muris, P., Huibers, M., Peeters, F., & Arntz, A. On the measurement of rumination: A psychometric evaluation of the ruminative response scale and the rumination on sadness scale in undergraduates. J. *Behav. Ther. Exp. Psychiatry.* **37**, 299-313 (2006).

65. Davey, G. C. A comparison of three worry questionnaires. *Behav. Res. Ther.* **31**, 51-56. (1993).

66. Hochman-Cohen, H. *The influence of emotional valence on word processing in the associative network.* (Unpublished Doctoral dissertation). (Ben-Gurion University of the Negev, 2008).

67. MacLeod, C., & McLaughlin, K. Implicit and explicit memory bias in anxiety: A conceptual replication. *Behav. Res. Ther.* **33**, 1-14 (1995).

68. Mathews, A., Mogg, K., May, J., & Eysenck, M. Implicit and explicit memory bias in anxiety. *J. Abnorm. Psychol.* **98**, 236; [10.1037/0021-843X.98.3.236](https://doi.org/10.1037/0021-843X.98.3.236) (1989).

69. Mathews, A., & MacLeod, C. Selective processing of threat cues in anxiety states. *Behav. Res. Ther.* **23**, 563-569 (1985).

70. Sharvit-Benbaji, H. *Data-base validation of depression and anxiety related words.* (Unpublished preliminary study for doctoral dissertation) (University of Haifa, 2015).

71. Lindquist, K. A., Wager, T. D., Kober, H., Bliss-Moreau, E., & Barrett, L. F. The brain basis of emotion: A meta-analytic review. *Behav. Brain. Sci.* **35**, 121-143 (2012).

72. Kron, A., Goldstein, A., Lee, D. H., Gardhouse, K., & Anderson, A. K. How are you feeling? Revisiting the quantification of emotional qualia. *Psychol. Sci.,* **24**, 1503-1511 (2013).

73. Plaut, D., & Frost, R. *The word-frequency database for printed Hebrew.* http://word- freq.mscc.huji.ac.il/ (2005).

74. Hindash, A. H. C., & Amir, N. Negative interpretation bias in individuals with depressive symptoms. *Cognit. Ther. Res.* **36**, 502-511 (2012).

75. Ogniewicz, A. S., Dugas, M. J., Langlois, F., Gosselin, P., & Koerner, N. An adapted word- sentence association paradigm for generalized anxiety and worry: Assessing interpretation bias. *J. Exp. Psychopathol.* **5**, 457-476 (2014).

76. Tottenham, N. et al. The NimStim set of facial expressions: Judgments from untrained research participants. *Psychiatry Res.* **168**, 242-249 (2009).

77. Lundqvist, D., Flykt, A., & Öhman, A. The Karolinska Directed Emotional Faces – KDEF. (CD ROM from Karolinska Institute, 1998).

78. Armstrong, T., & Olatunji, B. O. Eye tracking of attention in the affective disorders: A meta- analytic review and synthesis. *Clin. Psychol. Rev.* **32**, 704-723 (2012).

79. Leyman, L., De Raedt, R., Schacht, R., & Koster, E. H. Attentional biases for angry faces in unipolar depression. *Psychol. Med.* **37**, 393-402 (2007).

80. Pérez-Edgar, K. et al. Attention biases to threat and behavioral inhibition in early childhood shape adolescent social withdrawal. *Emotion,* **10**, 349; [10.1037/a0018486](https://doi.org/10.1037/a0018486) (2010).

81. Bradley, B. P. et al. Attentional biases for emotional faces. *Cogn. Emot.* **11**, 25-42 (1997).

82. Lang, P. J., Bradley, M. M., & Cuthbert, B. N. *International affective picture system (IAPS): Instruction manual and affective ratings. Technical report A-5.* <https://www2.unifesp.br/dpsicobio/adap/instructions.pdf> (2001).

83. Bradley, M. M., & Lang, P. J. Measuring emotion: The self-assessment manikin and the semantic differential. *J. Behav. Ther. Exp. Psychiatry.* **25**, 49-59 (1994).

84. Lang, P. J., Bradley, M. M., & Cuthbert, B. N. Motivated attention: Affect, activation, and action. In *Attention and orienting: Sensory and motivational processes*. (Lawrence Erlbaum Associates Publishers 1997).

85. Treutwein, B. Adaptive psychophysical procedures. *Vision Res.* **35**, 2503-2522 (1995).

86. Levitt, H. Transformed up‐down methods in psychoacoustics. *J. Acoust. Soc. Am.* **49**, 467-477 (1971).

87. Coles, M. E., & Heimberg, R. G. Memory biases in the anxiety disorders: Current status. *Clin. Psychol. Rev.* **22**, 587-627 (2002).

88. Schachter, D. L. Implicit memory: History and current status. *J. Exp. Psychol. Learn. Mem. Cogn.* **13**, 501-518 (1987).

89. Everaert, J., Duyck, W., & Koster, E. H. Attention, interpretation, and memory biases in subclinical depression: A proof-of-principle test of the combined cognitive biases hypothesis. *Emotion,* **14,** 331; [10.1037/a0035250](https://doi.org/10.1037/a0035250) (2014).

90. Andersen, S. M. The inevitability of future suffering: The role of depressive predictive certainty in depression. *Soc. Cogn,* **8**, 203; [10.1521/soco.1990.8.2.203](https://doi.org/10.1521/soco.1990.8.2.203) (1990).

91. Andersen, S. M., & Limpert, C. Future-event schemas: Automaticity and rumination in major depression. *Cognit. Ther. Res.* **25**, 311-333 (2001).

92. De Houwer, J. The extrinsic affective Simon task. *Exp. Psychol.* **50**, 77; [10.1026//1618- 3169.50.2.77](https://doi.org/10.1026/1618-3169.50.2.77) (2003).

93. Greenwald, A. G., McGhee, D. E., & Schwartz, J. L. Measuring individual differences in implicit cognition: The implicit association test. *J. Pers. Soc. Psychol.* **74**, 1464;  [10.1037/0022-3514.74.6.1464](https://doi.org/10.1037/0022-3514.74.6.1464) (1998).

94. Stöber, J., & Joormann, J. Worry, procrastination, and perfectionism: Differentiating amount of worry, pathological worry, anxiety, and depression. *Cognit. Ther. Res.* **25**, 49-60 (2001).

95. Oathes, D. J., Siegle, G. J., & Ray, W. J. Chronic worry and the temporal dynamics of emotional processing. *Emotion.* **11**, 101; [10.1037/a0021781](https://doi.org/10.1037/a0021781) (2011).
